# Aggression and Rabid Coyotes, Massachusetts, USA

**DOI:** 10.3201/eid1602.090731

**Published:** 2010-02

**Authors:** Xingtai Wang, Catherine M. Brown, Sandra Smole, Barbara G. Werner, Linda Han, Michael Farris, Alfred DeMaria

**Affiliations:** Massachusetts Department of Public Health, Boston, Massachusetts, USA

**Keywords:** Rabies, coyote, raccoon rabies virus variant, aggression, viruses, letter

**To the Editor:** In 1959, coyotes (*Canis latrans*) were found in only 3 Massachusetts towns, but by 2007, their population was estimated at 10,000 and they were present throughout the state, except on the islands of Martha’s Vineyard and Nantucket (*1*). The coyote is highly adaptable and readily tolerates living near humans (*2*). Because the raccoon rabies virus (RRV) variant is endemic to Massachusetts and spillover into the coyote population occurs (*3*), coyotes are a potential source of rabies exposure for humans. Rabies in coyotes has emerged in Massachusetts at the same time that coyote and human populations have increased. From 1985 through 2008, the Massachusetts Department of Public Health tested coyotes by following the standard direct fluorescent antibody testing protocol published by the Centers for Disease Control and Prevention (*4*).

Of the 111 coyotes submitted for rabies testing, 4 (3.6%) were unsatisfactory because of decomposed brain tissue. Of the remaining 107 coyotes, 10 (9.0%) were found to be rabid; strain typing confirmed all 10 to have had spillover RRV. Within each county, the time between the first identification of RRV in an animal and finding a rabid coyote within that county ranged from 558 to 4,857 days; median was 2,799 days. The long time before spillover from raccoon to coyote was detected suggests that coyotes might avoid rabid reservoir animals. The time lag may also be the result of the distinct ecologic niches of these animals; coyotes are the top predators in ecosystems, and raccoons are only 1 of several mesocarnivores.

The public health rabies surveillance system in the United States is passive and relies on interaction of humans or domestic animals with rabies vector species (*5*). Because a rabid wild animal would go untested if a human or domestic animal had not had potentially infectious contact with it, the 10 coyotes with confirmed rabies likely represented only some portion of all rabid coyotes in Massachusetts during the study period.

Among 97 nonrabid coyotes, 7 had reportedly been in contact with humans and domestic animals. Among the 10 rabid coyotes, 4 were reported to have been in contact with humans and domestic animals. The coyotes in contact with both were 8.6× more likely to be rabid than were those in contact with only 1 or the other (p<0.05).

Of the 111 coyotes submitted for testing, the reported circumstances of potentially infectious contact were as follows: capture (n = 5), dead animal contact (n = 1), fight (n = 11), handling (n = 26), provoked attack (n = 1), specimen preparation (n = 3), unprovoked attack on a human (n = 4), vicinity (n = 5), unknown (n = 47), and other (n = 8). The proportion of coyotes with positive rabies test results varied by type of contact as follows: fight (5/11), handling (1/26), unprovoked attack (2/4), and unknown (2/47). Likelihood of being rabid was 15.2× (p<0.0001) and 11.9× (p<0.05) higher for coyotes reported with fight contact and unprovoked attack behavior, respectively, than for coyotes with any other reported contact. Biting as type of contact was reported for 18 coyotes; positive rabies test results were found for 7. Coyotes that had reportedly bitten a person or domestic animal were 18.2× more likely to be rabid than were coyotes that had not (p<0.0001).

Of 11 coyotes for which aggression was reported, 6 had positive rabies test results (Table); aggressive animals were 27.6× more likely to be rabid than were those not reported to be aggressive (p<0.0001). These findings provide statistical support for anecdotal reports (from as early as 1958) of rabid coyotes showing aggression (*6*). The following were significantly associated with a positive rabies test result for submitted coyotes: having had contact both with humans and with domestic or companion animals, having attacked a person without provocation, having fought with dogs, and having bitten either a person or domestic animal. This association between aggressive behavior and a positive rabies test result is of particular concern because of coyotes’ relatively large size, their dramatically increased population, and their distribution throughout the state encompassing rural, suburban, and even urban areas. These factors increase the likelihood that a rabid animal will have the opportunity to interact with humans or their domestic animals, thus increasing the risk for rabies transmission.

**Table:** Reported signs of disease in 111 coyotes submitted for rabies testing, Massachusetts, USA, 1985–2008*

Clinical sign	Total no. (%)	DFA result		
		Positive	Negative	Unsatisfactory
Aggression				
No	100 (90.1)	4	92	4
Yes	11 (9.9)	6	5	0
Ataxia				
No	109 (98.2)	10	95	4
Yes	2 (1.8)	0	2	0
Disorientation				
No	94 (84.7)	8	83	3
Yes	17 (15.3)	2	14	1
Found dead				
No	96 (86.5)	8	86	2
Yes	15 (13.5)	2	11	2
Lethargy				
No	96 (86.5)	10	83	3
Yes	15 (13.5)	0	14	1
Paralysis				
No	106 (95.5)	10	92	4
Yes	5 (4.5)	0	5	0
Salivation				
No	108 (97.3)	9	95	4
Yes	3 (2.7)	1	2	0
Seizures				
No	109 (98.2)	9	96	4
Yes	2 (1.8)	1	1	0
Wound of unknown origin				
No	98 (88.3)	10	84	4
Yes	13 (11.7)	0	13	0

*DFA, direct fluorescence antibody; unsatisfactory, not tested because of decomposed brain tissue; no, not observed or unknown.

A limitation of our study is the fact that the descriptions of the circumstances surrounding human interactions with a coyote were provided by members of the general public. Coyotes are large and unfamiliar animals, and such reports are likely to be distorted by that unfamiliarity and the fear engendered by the interaction. Another limitation is that the reported clinical signs represent only a proportion of coyotes that were submitted for testing, usually those that had had potentially infectious contact with a human or domestic animal.

Data involving coyotes from other states would be of interest because of the ongoing spread of RRV and the variation in coyote habitat and population. As populations of coyotes in many areas of dense human population increase, the risk for rabies and aggressive behavior in coyotes presents challenges for public health and animal management.
